# Supplementary material for: Silicon isotopic signatures of granitoids support increased weathering of subaerial land 3.7 billion years ago
Source: Commun Earth Environ. 2025 May 16;6(1):382. doi: 10.1038/s43247-025-02337-7 (PMC12084155; doi:10.1038/s43247-025-02337-7)
Supplement: Supplementary file 2 — Supplementary Information [file 43247_2025_2337_MOESM2_ESM.pdf]

Supplementary Information to:

Silicon isotopic signatures of granitoids support increased weathering of subaerial land 3.7 billion years ago

Nicolas D. Greber, Madeleine E. Murphy, Julian-Christopher Storck, Jesse R. Reimink, Nicolas Dauphas, Paul S. Savage

Correspondence to: nicolas.greber@geneve.ch

Content:

- Supplementary Data 1 to 4
- Supplementary Figures 1 to 3
- Supplementary Notes 1 and 2
- Supplementary References

Supplementary Data:

Supplementary Data 1: Silicon and Ti isotope data of samples and geostandards measured in this study, as well as their published major element concentrations.

Supplementary Data 2: Titanium isotope data compilation used in this manuscript.

Supplementary Data 3: Silicon isotope data compilation used in this manuscript.

Supplementary Data 4: Input parameters for Si isotope modeling.

Supplementary Figures:

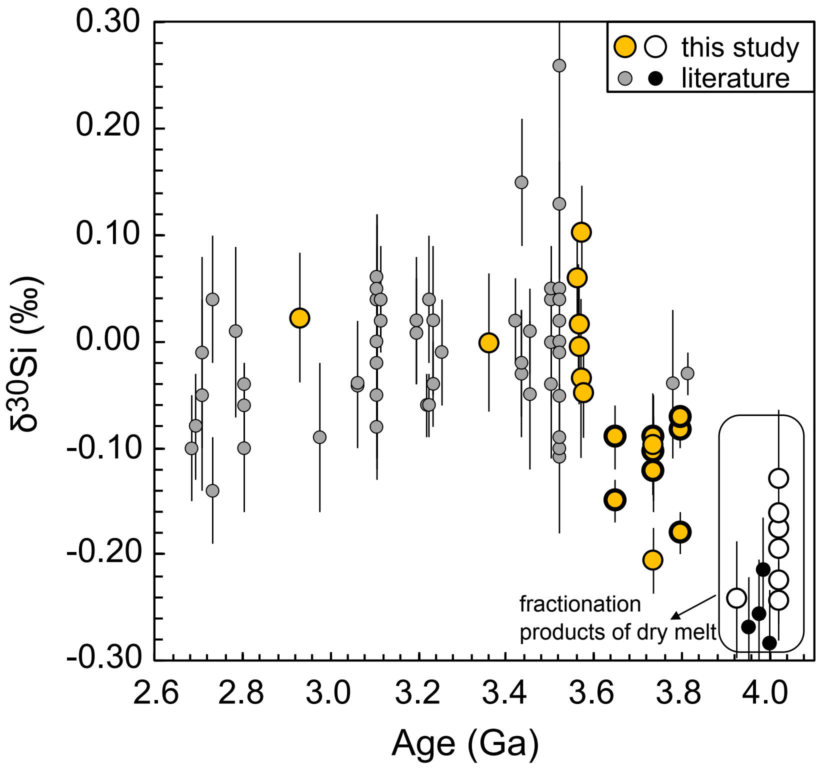

Supplementary Figure 1: Silicon isotopic compositions of granitoids over time (δ^{30}Si), i.e. the data are not corrected for the effect of magmatic differentiation. The same general trend is visible as in Figure 2 in the main manuscript. Literature data are from [1–4]. Samples from this study with a bold line are from Greenland, the others origin from the Acasta Gneiss. Data are given in supplementary Data 1 and 3.

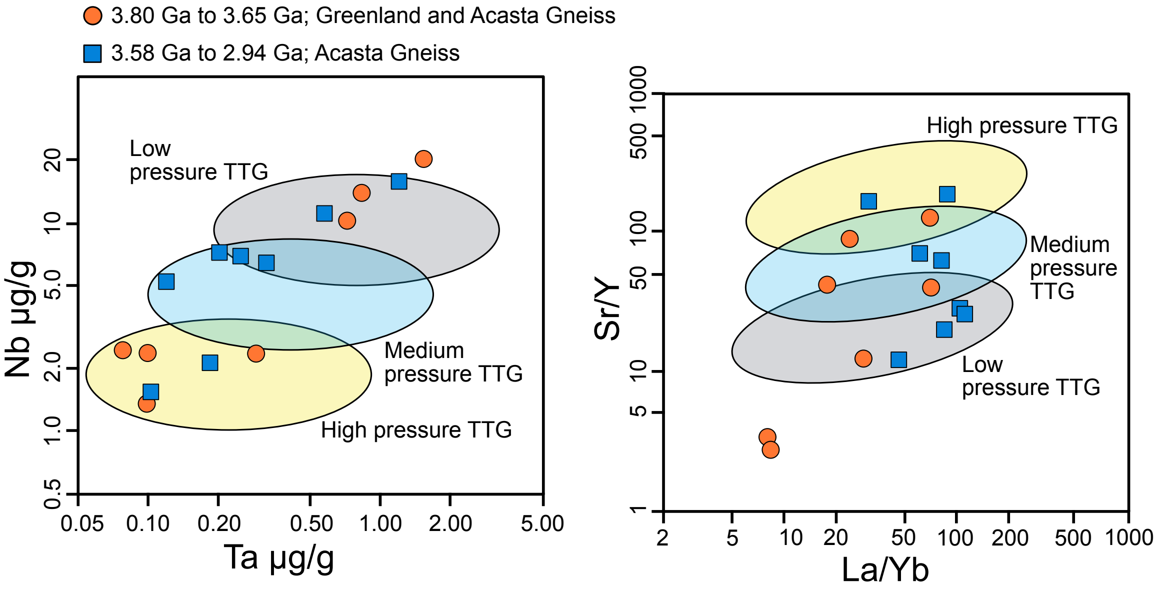

Supplementary Figure 2: Nb versus Ta, and Sr/Y versus La/Yb signatures of rocks originating from the melting of a hydrous mafic crust investigated in this study. Rocks from both age groups (younger and older than 3.60 Ga) cover the ranges of element signatures classically used to distinguish Archean low, medium and high pressure Tonalite-Trondjemite-Granodiorite (TTG) suites [5].

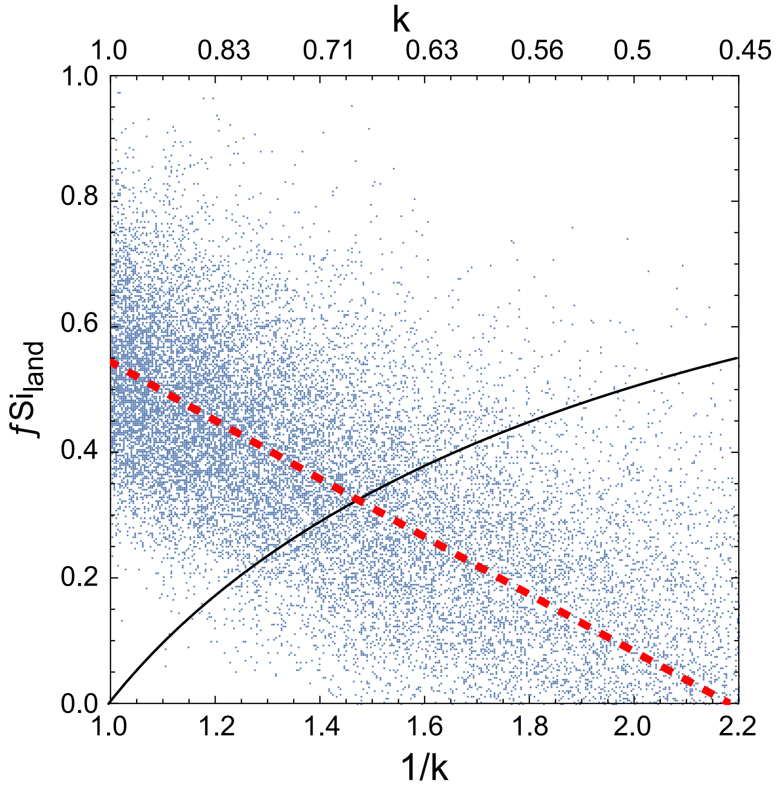

Supplementary Figure 3: Result of Model 3 (see supplementary Note 2 for details), with the dotted red line being a polynomial fit through the data produced by the Monte Carlo simulation (small blue points). Figure 3B in the main manuscript is based on this modeling result. It correlates the calculated fraction of the Si flux from the weathering of land ($f\mathrm{Si}_{\mathrm{land}}$) to the ocean, based on the Si isotopic composition of the measured granitoids, with the changing proportion of silica ($1/k = \frac{p_{<3.6}}{p_{>3.6}}$) added to their melting sources. The black line represents the situation where the increase in silica in the melting source of the granitoids after 3.60 Ga is identical to the fraction of the Si flux to the ocean from the weathering of land. 1/k (x-axis) expresses the relative increase in the added silica to the melting source of granitoids younger than 3.60 Ga compared to older ones. At a value of 1, the amount of silica added to the melting source stays identical, at a value of 2, it doubles. Note that for a value of 2.2 times more silica added to the melting source of the granitoids, $f\mathrm{Si}_{\mathrm{land}}$ is 0. This means that the additional silica added to the ocean had a different isotopic composition from what we assumed to be the discharge from the weathering of emerged crust. This could mean either that the additional Si did not derive from the erosion of land, or that the Si isotopic composition delivered by land to the ocean had a much lower Si isotopic composition, more akin to that of the unweathered continental crust. We prefer the latter, as increasing seawater silica in the melting source of granitoids likely requires a higher flux of Si to the ocean. Hydrothermal discharge likely decreased, and not increased over time [6–8], and aside from Si input from emerged land, no other major Si flux to the ocean is currently known.

Supplementary Notes 1

Sample description

Acasta Gneiss samples

All Acasta Gneiss samples analyzed here have been extensively described in both their petrology and isotope geochemistry, including whole rock elemental geochemistry [9,10], zircon oxygen and hafnium isotope data [9–11], extinct isotope data [12] and Ti isotope data [13]. Our measured tonalites and granitoids span an age range from 4.02 to 2.94 Ga. Radiogenic isotope composition and trace elements imply that their melting source and mechanism of formation changed over time [12]. The 4.02 Ga rocks are 'icelandite'-like in elemental composition and were likely formed by high degrees of shallow fractionation starting from an anhydrous basaltic melt. In contrast, the 3.75 Ga tonalites were generated by partial melting of hydrated Hadean mafic crust in a shallow melting environment. Sample with ages ≤3.60 Ga formed by melting of young, hydrated mafic crust with almost chondritic Hf isotopes at greater depth than the 3.75 Ga tonalites.

West Greenland samples

Eoarchaean TTG orthogneiss samples (all having prefix 155) were collected from the Itsaq Gneiss Complex of southern West Greenland by Stephen Moorbath and colleagues [14], and they have been well-characterized by a number of workers [15–18]. Most of the samples are from the Nuuk region, including several from the Godthåbsfjord area and one sample from the Qilanngaarsuit area (155735). Three additional samples come from Amîtsoq gneisses of the Isua Supracrustal Belt dome (155766, 155768 and 155774). The Itsaq gneisses formed from a TTG-type protolith in the Eoarchaean and were subsequently metamorphosed to the granulite facies, and they show a limited range in textures and compositions, mostly comprising granitic, "speckled" dioritic, and banded gneisses. These TTG samples largely constitute plagioclase and K-feldspar, quartz, and biotite [14], and their Pb/Pb whole rock ages range from 3800 ± 120 Ma [19] to 3654 ± 73 Ma [20].

Supplementary Notes 2

Mass balance models

The errors are calculated as the 2SD of a Monte Carlo simulation for error propagation (20'000 cycles), unless the simulated data distribution is skewed. In the latter case we report the 2.5% to 97.5% percentile range as error estimates. For our model, we use all granitoids that sourced from the melting of hydrated basalts (i.e. excluding the rocks that derive from fractionation of dry melts). Some of the published granitoids exhibit high SiO_2 concentrations (up to 86 wt%), exceeding the typical values for highly felsic rocks such as rhyolites (i.e., 77 wt%). Generally, samples with SiO_2 concentrations above 77 wt% display greater variability in

$\Delta^{30}Si$. This may indicate that normalization for the effect of fractional crystallization on Si isotopes is less accurate at such elevated SiO_2 concentrations. However, excluding these data does not alter the outcomes of our model. All needed input variables are given in supplementary Data 4.

Model 1; static Si isotopic composition of silica added to the seafloor

The offset in the Si isotopic composition of Archean granitoids compared to modern ones ($\Delta^{30}Si$) likely equals the offset between a non silicified basalt and the silicified basalt that partially melts [3]. In this case, $\Delta^{30}Si$ is defined as:

$$\Delta^{30}Si = (p * \delta^{30}Si_{silica} + (1\text{-}p) * \delta^{30}Si_{basalt}) - \delta^{30}Si_{basalt} \quad \text{(eq. S1),}$$

With *p* being the proportion of silica added, $\delta^{30}Si_{silica}$ being the isotopic composition of the silicifying material, and $\delta^{30}Si_{basalt}$ is the isotopic composition of the non silicified basalt (-0.29 ± 0.08‰ [21]). Writing this equation for the time intervals before and after 3.60 Ga yields:

$$\Delta^{30}Si_{Gr<3.6} = (p_{<3.6} * \delta^{30}Si_{silica<3.6} + (1\text{-}p_{<3.6}) * \delta^{30}Si_{basalt}) - \delta^{30}Si_{basalt} \quad \text{(eq. S1a),}$$

$$\Delta^{30}Si_{Gr>3.6} = (p_{>3.6} * \delta^{30}Si_{silica>3.6} + (1\text{-}p_{>3.6}) * \delta^{30}Si_{basalt}) - \delta^{30}Si_{basalt} \quad \text{(eq. S1b),}$$

with $\Delta^{30}Si_{Gr<3.6}$ being the $\Delta^{30}Si$ value of granitoids younger than 3.60 Ga (i.e. +0.160 ± 0.018‰; 2SE, n= 67), and $\Delta^{30}Si_{Gr>3.6}$ being the $\Delta^{30}Si$ value of granitoid between 3.82 Ga to 3.60 Ga (i.e. +0.075 ± 0.030‰; 2SE, n=12). Given that in this model we assume that $\delta^{30}Si_{silica<3.6}$ equals $\delta^{30}Si_{silica>3.6}$ we can combine and rewrite these equations to:

$$\frac{\Delta^{30}Si_{Gr<3.6}}{\Delta^{30}Si_{Gr>3.6}} = \frac{p_{<3.6}}{p_{>3.6}} \quad \text{(eq. S2).}$$

Thus, when assuming that the Si isotopic composition of silica added to the seafloor did not change, we require that around 2.2 times more silica was added to the melting source of the granitoids younger than 3.60 Ga, compared to those older than 3.60 Ga. The result of the Monte Carlo simulation is skewed, with the 2.5% and 97.5% percentile yielding values of 1.5 and 3.5, respectively. Thus, if the additional Si added to ocean is due to the onset of continental weathering, the Si flux from land would make up around 55% of the total input.

Model 2; static proportion of silica added to the melting source of the granitoids

For this model, where we assume that the proportion of silica added to the melting source of the granitoids did not change, we can use equation S1 to calculate *p* in the case the $\delta^{30}Si_{silica}$ is known (eq. S3), or one can calculate $\delta^{30}Si_{silica}$ in the case *p* is known (eq. S4).

$$p = -\frac{\Delta^{30}Si}{\delta^{30}Si_{basalt} - \delta^{30}Si_{silica}} \quad \text{(eq. S3)}$$

$$\delta^{30}Si_{silica}=\frac{\Delta^{30}Si+\delta^{30}Si_{basalt}*p}{p} \qquad \text{(eq. S4)}$$

In our case, we can calculate *p* with equation S3 for the granitoids that are younger than 3.60 billion years ($\Delta^{30}Si_{Gr<3.6}$ = +0.160 ± 0.018‰; 2SE, n=67), where we have information on the Si isotopic composition of the silica added to their melting source ($\delta^{30}Si_{silica<3.6}$), estimated to be between +0.9‰ to +1.5‰ [3]. Assuming *p* stays constant, we can then calculate through equation S4 the $\delta^{30}Si_{silica>3.6}$ of the silica added to the melting source of the granitoids older than 3.60 billion years ($\Delta^{30}Si_{Gr>3.6}$ = +0.075 ± 0.030‰; 2SE, n=12). The difference in the isotopic composition of the silica added to the melting source of the >3.60 Ga and <3.60 Ga granitoids (ΔSi_{silica}) is then defined as:

$$\Delta Si_{silica}=\delta^{30}Si_{silica<3.6}-\delta^{30}Si_{silica>3.6} \qquad \text{(eq. S5)}$$

Combining equations S3, S4 and S5 leads to:

$$\Delta Si_{silica}=\frac{(\delta^{30}Si_{basalt}-\delta^{30}Si_{silica<3.6})*(\Delta^{30}Si_{Gr>3.6}-\Delta^{30}Si_{Gr<3.6})}{\Delta^{30}Si_{Gr<3.6}} \qquad \text{(eq. S6).}$$

If the ΔSi_{silica} is the result of a heavy $\delta^{30}Si$ flux to the ocean ($\delta^{30}Si_{input}$) due to the onset of weathering and erosion of land, then the fraction of the Si flux from land (fSi_{land}) to the ocean can be described as:

$$\delta^{30}Si_{input}=(\delta^{30}Si_{hyd}+\Delta Si_{silica})=\delta^{30}Si_{land}*fSi_{land}+(1-fSi_{land})*\delta^{30}Si_{hyd} \qquad \text{(eq. S7)}$$

Solving this equation for fSi_{land} yields:

$$fSi_{land}=-\frac{\Delta Si_{silica}}{\delta^{30}Si_{hyd}-\delta^{30}Si_{land}} \qquad \text{(eq. S8).}$$

Here, $\delta^{30}Si_{hyd}$ and $\delta^{30}Si_{land}$ are the Si isotopic compositions of the oceanic inputs through hydrothermalism and dissolved Si from the erosion of land, respectively. The isotopic composition of the hydrothermal input ($\delta^{30}Si_{hyd}$) is estimated at -0.30 ± 0.14‰ (error as shown in Rahman and Trower [22]) and that of the dissolved Si ($\delta^{30}Si_{land}$) at +1.210 ± 0.078 (2SE, n=372) [22]. Substituting ΔSi_{silica} from equation S6 into equation S8 leads to:

$$fSi_{land}=\frac{(\delta^{30}Si_{basalt}-\delta^{30}Si_{silica<3.6})*(\Delta^{30}Si_{Gr<3.6}-\Delta^{30}Si_{Gr>3.6})}{\Delta^{30}Si_{Gr<3.6}*(\delta^{30}Si_{hyd}-\delta^{30}Si_{land})} \qquad \text{(eq. S9).}$$

Using the input values given in supplementary Data 4 yields for fSi_{land} a value of 0.53 ± 0.26.

The model is based on two fundamental premises. (i) We imply that the change in the isotopic composition of the silica added to the melting source of the granitoids is equal to the change in seawater δ^{30}Si, which in turn is directly connected to the change in the total δ^{30}Si flux to the ocean due to the onset of the erosion of land. (ii) We assume that the Δ^{30}Si value of +0.075 ± 0.030‰ (2SE, n=12) of the granitoids aged between 3.82 Ga and 3.65 Ga does not require a continental silica flux to the ocean. This assumption is to date substantiated by equation S4, that allows us to calculate the $\delta^{30}Si_{silica>3.6}$, representing the Si isotopic composition of silica added to the metabasalts. The best estimates for $\delta^{30}Si_{silica>3.6}$ yield between +0.21‰ (for $\delta^{30}Si_{silica<3.6}$ = 0.9‰) and +0.46‰ (for $\delta^{30}Si_{silica<3.6}$ = 1.5‰). Such values can be attributed to Si sinks in the ocean, specifically amorphous silica precipitation and reverse weathering, which exhibit isotope fractionations with seawater of around -0.5‰ and -2.0‰, respectively [23–25]. Given that the Si isotopes in the oceanic system were at steady-state (i.e. $\delta^{30}Si_{input} = \delta^{30}Si_{output} = \delta^{30}Si_{hyd}$) a seawater δ^{30}Si of +0.21‰ to +0.46‰ can be explained by a Si sink comprising 80-90% amorphous silica precipitation and 10-20% reverse weathering, for instance. This is in agreement with estimates for the reverse weathering sink in the early Archean, albeit with large errors [26–28].

Model 3: Combining higher Si flux with changing isotopic composition

Given that the erosion of land probably resulted in an increased Si flux to the ocean that was also isotopically heavier, the two endmember models above likely overestimate the contribution of continental silicon. Thus, we also developed a model that combines the effects of an isotopically heavier and increased Si flux. For this model we use again the two premises as above; (i) the change in the isotopic composition of the silica added to the melting source of the granitoids is equal to the change in seawater δ^{30}Si due to the onset of the erosion of land, and (ii) we assume that the Δ^{30}Si value of +0.075 ± 0.030‰ (2SE, n=12) of the granitoids aged between 3.82 Ga and 3.65 Ga does not require a continental silica flux to the ocean.

To adjust the model above, we add a parameter *k* that describes the difference in the silica added to melting source of granitoids before and after 3.60 Ga:

$$k = \frac{p_{>3.6}}{p_{<3.6}} \quad \text{(eq. S10).}$$

We then rewrite equation S1 to:

$$\Delta^{30}Si' = \left(p * ((1-k) * \delta^{30}Si_{hyd} + k * \delta^{30}Si_{land}) + (1-p) * \delta^{30}Si_{basalt}\right) - \delta^{30}Si_{basalt} \quad \text{(eq. S11),}$$

and adjust equation S9 to:

$$f Si_{land}' = \frac{(\delta^{30}Si_{basalt} - \delta^{30}Si_{silica<3.6}) * (-\Delta^{30}Si_{Gr>3.6} + \Delta^{30}Si_{Gr<3.6} * k)}{\Delta^{30}Si_{Gr<3.6} * (\delta^{30}Si_{hyd} - \delta^{30}Si_{land}) * k} \quad \text{(eq. S12).}$$

If we state that the change in the proportion of silica added to the melting source is identical to the increased Si flux from the onset of the weathering of land, then the parameter k is related to $f\mathrm{Si}_{\mathrm{land}}'$ through:

$$1 - k = 1 - \frac{p_{>3.6}}{p_{<3.6}} = \frac{p_{<3.6} - p_{>3.6}}{p_{<3.6}} = f\mathrm{Si}_{\mathrm{land}}' \qquad \text{(eq. S13).}$$

We can now substitute k for $1 - f\mathrm{Si}_{\mathrm{land}}'$ in equation S12 and solve for $f\mathrm{Si}_{\mathrm{land}}'$ (see supplementary Figure 3 and Figure 3 in the main manuscript), which yields 0.32 ± 0.15. However, it is currently not precisely known how the proportion of silica added to the melting source (k) and the increased flux of Si to the ocean are connected. We can thus also use equation S12 to show how $f\mathrm{Si}_{\mathrm{land}}'$ changes at different k, which is illustrated in supplementary Figure S3, which shows the result of the Monte Carlo modeling of equation S12 by picking randomly a real number for k between 1 and 0.45 (the realistic range given by Model 1). This allows to display the range of combinations between increased Si in the melting source of the granitoids and the Si flux from the weathering of emerged land that can explain the Si isotopic compositions of the granitoids.

Supplementary References:

1. Deng, Z. *et al.* An oceanic subduction origin for Archaean granitoids revealed by silicon isotopes. *Nat. Geosci.* **12**, 774–778 (2019).
2. André, L. *et al.* Early continental crust generated by reworking of basalts variably silicified by seawater. *Nat. Geosci.* **12**, 769–773 (2019).
3. Murphy, M. E. *et al.* Silicon isotopes in an Archaean migmatite confirm seawater silicification of TTG sources. *Geochim. Cosmochim. Acta* **368**, 34–49 (2024).
4. Zhang, Q. *et al.* No evidence of supracrustal recycling in Si-O isotopes of Earth's oldest rocks 4 Ga ago. *Sci. Adv.* **9**, 1–10 (2023).
5. Moyen, J. F. & Martin, H. Forty years of TTG research. *Lithos* **148**, 312–336 (2012).
6. Tajika, E. & Matsui, T. Evolution of terrestrial proto-CO2 atmosphere coupled with thermal history of the earth. *Earth Planet. Sci. Lett.* **113**, 251–266 (1992).
7. O'Neill, C., Lenardic, A., Höink, T. & Coltice, N. Mantle Convection and Outgassing on Terrestrial Planets. *Comp. Climatol. Terr. Planets* 473–486 (2013) doi:10.2458/azu_uapress_9780816530595-ch19.
8. Lowell, R. P. & Keller, S. M. High-temperature seafloor hydrothermal circulation over geologic time and archean banded iron formations. *Geophys. Res. Lett.* **30**, 1–4 (2003).
9. Reimink, J. R., Chacko, T., Stern, R. A. & Heaman, L. M. Earth's earliest evolved crust generated in an Iceland-like setting. *Nat. Geosci.* **7**, 529–533 (2014).
10. Reimink, J. R., Chacko, T., Stern, R. A. & Heaman, L. M. The birth of a cratonic nucleus: Lithogeochemical evolution of the 4.02-2.94 Ga Acasta Gneiss Complex. *Precambrian Res.* **281**, 453–472 (2016).
11. Reimink, J. R. *et al.* No evidence for Hadean continental crust within Earth's oldest evolved rock unit. *Nat. Geosci.* **9**, 777–780 (2016).
12. Reimink, J. R. *et al.* Petrogenesis and tectonics of the Acasta Gneiss Complex derived from integrated petrology and 142Nd and 182W extinct nuclide-geochemistry. *Earth Planet. Sci. Lett.* **494**, 12–22 (2018).
13. Aarons, S. M. *et al.* Titanium isotopes constrain a magmatic transition at the Hadean-Archean boundary in the Acasta Gneiss Complex. *Sci. Adv.* **6**, 1–9 (2020).
14. Moorbath, S., O'Nions, R. K., Pankhurst, R. J., Gale, N. H. & McGregor, V. R. Further Rubidium-Strontium Age Determinations on the Very Early Precambrian Rocks of the Godthaab District, West Greenland. *Nat. Phys. Sci.* **240**, 78–82 (1972).
15. O'Nions, R. K. & Pankhurst, R. J. Rare-earth element distribution in Archaean gneisses and anorthosites, Godthåb area, West Greenland. *Earth Planet. Sci. Lett.* **22**, 328–338 (1974).
16. Kamber, B. S., Ewart, A., Collerson, K. D., Bruce, M. C. & McDonald, G. D. Fluid-mobile trace element constraints on the role of slab melting and implications for Archaean crustal growth models. *Contrib. to Mineral. Petrol.* **144**, 38–56 (2002).

17. Caro, G., Bourdon, B., Birck, J. L. & Moorbath, S. High-precision 142Nd/144Nd measurements in terrestrial rocks: Constraints on the early differentiation of the Earth's mantle. *Geochim. Cosmochim. Acta* **70**, 164–191 (2006).

18. Fedo, C. M., Whitehouse, M. J. & Kamber, B. S. Geological constraints on detecting the earliest life on Earth: A perspective from the Early Archaean (older than 3.7 Gyr) of southwest Greenland. *Philos. Trans. R. Soc. B Biol. Sci.* **361**, 851–867 (2006).

19. Moorbath, S., O'Nions, R. K. & Pankhurst, R. J. The evolution of early precambrian crustal rocks at Isua, West Greenland - Geochemical and isotopic evidence. *Earth Planet. Sci. Lett.* **27**, 229–239 (1975).

20. Kamber, B. S. & Moorbath, S. Initial Pb of the Amitsoq gneiss revisited: Implication for the timing of early Archaean crustal evolution in West Greenland. *Chem. Geol.* **150**, 19–41 (1998).

21. Savage, P. S., Georg, R. B., Armytage, R. M. G., Williams, H. M. & Halliday, A. N. Silicon isotope homogeneity in the mantle. *Earth Planet. Sci. Lett.* **295**, 139–146 (2010).

22. Rahman, S. & Trower, E. J. Probing surface Earth reactive silica cycling using stable Si isotopes: Mass balance, fluxes, and deep time implications. *Sci. Adv.* **9**, 1–13 (2023).

23. Hughes, H. J., Sondag, F., Santos, R. V., André, L. & Cardinal, D. The riverine silicon isotope composition of the Amazon Basin. *Geochim. Cosmochim. Acta* **121**, 637–651 (2013).

24. Ehlert, C. *et al.* Stable silicon isotope signatures of marine pore waters – Biogenic opal dissolution versus authigenic clay mineral formation. *Geochim. Cosmochim. Acta* **191**, 102–117 (2016).

25. André, L., Monin, L. & Hofmann, A. The origin of early continental crust: New clues from coupling Ge/Si ratios with silicon isotopes. *Earth Planet. Sci. Lett.* **582**, 117415 (2022).

26. Trower, E. J. & Fischer, W. W. Precambrian Si isotope mass balance, weathering, and the significance of the authigenic clay silica sink. *Sediment. Geol.* **384**, 1–11 (2019).

27. Isson, T. T. & Planavsky, N. J. Reverse weathering as a long-term stabilizer of marine pH and planetary climate. *Nature* **560**, 471–475 (2018).

28. Krissansen-Totton, J. & Catling, D. C. A coupled carbon-silicon cycle model over Earth history: Reverse weathering as a possible explanation of a warm mid-Proterozoic climate. *Earth Planet. Sci. Lett.* **537**, 116181 (2020).
