# Supplementary material for: Silicon isotopic signatures of granitoids support increased weathering of subaerial land 3.7 billion years ago
Source: Commun Earth Environ. 2025 May 16;6(1):382. doi: 10.1038/s43247-025-02337-7 (PMC12084155; doi:10.1038/s43247-025-02337-7)
Supplement: Supplementary file 3 — Description of Additional Supplementary Files [file 43247_2025_2337_MOESM3_ESM.docx]

**Description of Additional Supplementary Files**

**File name:** Supplementary Data 1

**Description:** Silicon and Ti isotope data of samples and geostandards measured in this study, as well as their published major element concentrations.

**File name:** Supplementary Data 2

**Description:** Titanium isotope data compilation used in this manuscript.

**File name:** Supplementary Data 3

**Description:** Silicon isotope data compilation used in this manuscript.

**File name:** Supplementary Data 4

**Description:** Input parameters for Si isotope modeling.
